# Empty follicle syndrome following GnRH agonist stimulation, in a patient with PCOS treated with HCG rescue protocol, resulting in 3PN zygote formation: a case report

**DOI:** 10.5935/1518-0557.20230051

**Published:** 2024

**Authors:** Nasrin Saharkhiz, Nazanin Hajizade, Mahsa Kazemi, Samaneh Esmaeili, Bahareh Karimi

**Affiliations:** 1 Department of Obstetrics and Gynecology, School of Medicine, Shahid Beheshti University of Medical Sciences, Tehran, Iran; 2 Preventative Gynecology Research Center, Shahid Beheshti University of Medical Sciences, Tehran, Iran; 3 Department of Biology and Anatomical Sciences, School of Medicine, Shahid Beheshti University of Medical Sciences, Tehran, Iran; 4 Department of Cell Biology ana Anatomical Science, Faculty of Medicine, Shahid Beheshti University of Medical Science, Tehran, Iran

**Keywords:** empty follicle syndrome, tripronuclear embryo, ICSI

## Abstract

Empty follicle syndrome is a rare condition characterized by failure to retrieve oocytes despite repeated careful aspiration of mature precursor follicles during controlled ovarian stimulation. This report presents a case of empty follicle syndrome in a patient with polycystic ovary syndrome using a gonadotropin-releasing hormone agonist as a trigger for final oocyte maturation. No oocytes were retrieved from the right ovary and the procedure was discontinued. The patient was administered an injection with 10,000 units of HCG and 3 oocytes were obtained after 24 hours. All oocytes were mature (MII); fertilization was performed with sperm from the patient’s husband resulting in 3PN zygotes. The formation of 3PN zygotes from ICSI might be due to oocyte cytoplasmic disorders caused by long-term exposure to gonadotropins and increased duration of stimulation. Although our patient had false empty follicle syndrome and the hCG rescue protocol led to the retrieval of oocytes, the oocytes were not of good quality. As previously described, empty follicle syndrome is not a predictor of success in subsequent cycles. Our patient’s next cycle was uneventful.

## INTRODUCTION

Empty follicle syndrome (EFS) is defined as the failure to retrieve oocytes after ovarian stimulation and follicle aspiration in IVF and ICSI cycles, despite the presence of apparently normal ovarian follicles and appropriate estradiol levels (Pujalte *et al*., 2023). The incidence of this syndrome has been reported to be 0.6-7.0% (Mesen *et al*., 2011; Baum *et al*., 2012). The mechanism responsible for this syndrome is unknown. However, factors such as premature ovarian atresia caused by inefficient folliculogenesis, ovarian aging in elderly women, and some ovulation stimulants are considered to be factors involved in this syndrome (Singh *et al*., 2018).

Human chorionic gonadotropin (hCG), due to its similarity to luteinizing hormone (LH), is used to induce final oocyte maturation after ovarian stimulation in all IVF and ICSI cycles. But hCG, due to its longer half-life and prolonged luteotrophic effect, can lead to ovarian hyperstimulation syndrome (OHSS), especially in patients with polycystic ovary syndrome (PCOS). In recent years, gonadotropin-releasing hormone agonist (GnRHa) has become an alternative to hCG in the prevention of OHSS in PCOS patients due to the more widespread use of GnRH antagonist (GnRHa) protocols. But the molecular structure, place and mechanism of action of these two hormones in increasing LH are different from each other (Humaidan *et al*., 2010). The development of EFS in PCOS patients following the use of GnRHa can happen for several reasons (Deepika *et al*., 2018) but in some cases, such as the one reported herein, it can be resolved with the hCG rescue protocol.

Triploidy is one of the most common chromosomal abnormalities. At the cellular level, triploidy is characterized by the presence of three (3n) rather than two (2n) haploid chromosome complements (Figueira *et al*., 2011). Current data suggest that 3PN formation in ICSI occurs as a consequence to the lack of extrusion of the second polar body. Some studies suggest that severe sperm abnormalities, oocyte aging in older women, and high response to gonadotropins can contribute to this process. Also, adjustable variables of ovarian stimulation, including starting dose, total amount of gonadotropins administered, and number of days of stimulation are each independent predictors of 3PN formation (Rosen *et al*., 2006).

The purpose of the present study is to describe a case of empty follicle syndrome, in which, despite the rescue procedure, all the resulting embryos were 3PN.

## CASE PRESENTATION

A 22-year-old healthy woman, gravida zero, and her husband were referred to our clinic (Infertility and IVF Center of Taleghani Hospital) because of combined primary infertility. They had been married for five years and suffering from infertility for three years.

The wife was diagnosed with PCOS based on oligomenorrhea, hyperandrogenism (acne and mild hirsutism), and ultrasound findings (PCO Morphology). Her BMI was 26.7kg/m^2^. Her anti-Müllerian hormone (AMH) level was 9.6 ng/mL.

Her husband, who had bilateral varicocelectomy six years earlier, smoked occasionally. Sperm volume, count, and motility were just within the normal range, but morphology was affected with a lower-than-normal range according to guidelines for ICSI. DFI was 14%. The couple had a history of six failed IUI cycles.

After several months of life style modification, COS (controlled ovarian stimulation) started due to PCO and male factor (teratospermia). On day 2 of the menstrual cycle, recombinant FSH (Cinnalef…) 150 IU was administered. On the CD2 (cycle day 2), the patient had one 13-mm follicle in the left ovary and multiple follicles measuring 10 mm or less. Her prescription of Cinnalef was decreased to 75 IU and 75 IU uHMG (urinary Human Menopausal Gonadotropin) was added. A GNRH antagonist (Cetrotide…) was started on CD6. On CD8, due to numerous dominant follicles measuring 16-18mm in both ovaries, she was given Gnanis (Variopeptyl 0.3mg) as a trigger to protect her from OHSS. Twelve hours after GNRH agonist injection, serum LH was 35IU/l. On CD10, ovum pickup (OPU) was performed. The right ovary had no oocytes, despite multiple dominant follicles on TVS. Fluid of the cul de sac was tested, no oocyte was there either. In spite of correct administration and drug dosage, she had empty follicular syndrome (EFS) and OPU was discontinued.

On the same day, a rescue dose of HCG (10000 units) was administered. Twenty-four hours later, OPU was performed in the left ovary. Several follicles were retrieved, and only three M2 oocytes were collected. (One was amorph and one was one had a fragmented polar body, cytoplasm of all of the oocytes was granulated). ICSI was performed with abnormal sperm (abnormal morphology was zero percent). Three 3PN (pronuclear) zygotes were formed. They were not frozen.

Six months later a second COS cycle was started. On CD2, recombinant HMG (Pergoveris^®^, combination of 150 IU FSH and 75 IU LH) was administered. On D5, the left ovary had two follicles measuring 12 and 14mm. The right ovary had multiple follicles which the largest measuring 12mm. GnRH agonist (Cetrotide) was started on CD6. On D7, since both ovaries contained multiple follicles above 16 mm, a GNRH agonist trigger was administered. Twelve hours after the GNRH Agonist injection, serum LH was 143IU/l. OPU was performed on CD10. A total of eight oocytes from the left ovary and seven from the right ovary were retrieved. One oocyte was degenerated, and 14 were M2. All had wide and granulated cytoplasms. Three also had a thin zona pellucida. ICSI was performed with normal sperm. Finally, nine moderate to low 3-day embryos (26 B.18D, 16C, 28B, 15 C, 28C) were frozen and the patient was a candidate for FET.

## DISCUSSION

The success of oocyte retrieval is influenced by several important factors: the type of trigger, the dose of the trigger and the time interval from trigger injection to oocyte retrieval (Song & Sun, 2019).

GnRHa has emerged as the stimulant of choice in patients with polycystic ovary syndrome, hyperresponders, and donors, as it reduces the risk of OHSS due to its short half-life (60-120 minutes) (Türkgeldi *et al*., 2015; Engmann *et al*., 2008). GnRH agonists act on the pituitary gland and release LH and FSH. Therefore, the inability of the pituitary gland to release gonadotropins or a deficiency in one of the mediators/receptors on the ovary can hinder oocyte maturation and lead to EFS (Deepika *et al*., 2018).

EFS is described in two ways: genuine EFS (GEFS) and false EFS (FEFS). The genuine type is related to inherent ovary dysfunction and the false type is related to drug problems (Stevenson & Lashen, 2008).

GEFS has been linked to conditions such as inefficient folliculogenesis (especially in patients with PCOS) (Ben-Shlomo *et al*., 1991), dysfunction of granulosa cells (Inan *et al*., 2006), disorder of oocyte growth and maturation genetics, and ovarian aging (Yuan *et al*., 2017). Patients with GEFS are unlikely to respond to the hCG rescue protocol (Deepika *et al*., 2018).

FEFS has been reported to occur after GnRHa stimulation in patients with PCOS following failure to induce an optimal endogenous LH surge and/or progesterone surge (Stevenson & Lashen, 2008). Potential reasons include hypothalamic-pituitary-ovarian (HPO) axis disorders, human errors in timing, preparation or administration of stimulant medication (Reichman *et al*., 2010), manufacturing problems, and abnormal biological activity of some commercially available GnRHa classes in vivo (Kummer *et al*., 2013). In cases of FEFS, a timely salvage stimulus can lead to successful cycle outcomes in selected cases (Deepika *et al*., 2018).

In our case, rescue stimulus with 10,000 units of HCG led to the retrieval of three oocytes, but the problem was that all the oocytes were 3PN after sperm was injected into them; as a result, there were no suitable embryos for transfer. Macas *et al*. (2001) evaluated the formation of 3PN zygotes in cases of ICSI in which the man had severe sperm abnormalities. They found that 33% of triploids resulted from diploid sperm (Figueira *et al*., 2011). Our patient’s husband had teratozoospermia, but since the patient had no 3PN zygotes in the next cycle, it can be assumed that the formation of 3PN zygotes probably indicates dysfunction of the oocyte and its cytoplasm.

Previous studies have shown that the mechanism of 3PN zygote formation after ICSI occurs mainly due to failed expulsion of the second polar body in meiosis II. This condition may occur due to a damaged metaphase plate or oocyte cytoskeleton after abnormal spindle formation. Researchers have suggested that specific stimulation parameters such as total gonadotropin dose, E2 level and increased oocyte production, number of stimulation days, trigger day, and in vitro oocyte aging in ICSI rescue protocol may induce triploid zygotes (Rosen *et al*., 2006).

It is still debatable which of the cases can definitely predict the development of 3PN zygotes. In our case, change in the trigger day and the increase in the number of stimulation days are potential causes for the development of 3PN zygotes.

## CONCLUSION

An infertile patient with polycystic ovary syndrome was referred to the infertility department at Taleghani Hospital, Shahid Beheshti University of Medical Sciences. She underwent ART with ovarian stimulation and developed empty follicle syndrome. With the hCG rescue protocol, we were able to obtain oocytes, but unfortunately, all developed into 3PN zygotes after ICSI. The rescue protocol might not always work and oocytes may develop into 3PN zygotes due to changes in the trigger day. Therefore, in order to avoid cases in which patients develop 3PN zygotes after ICSI, we must pay attention to the stimulation and trigger protocol in the next cycle.
